# The relationship between physical activity and lymphoma: a systematic review and meta analysis

**DOI:** 10.1186/s12885-020-07431-x

**Published:** 2020-10-06

**Authors:** Gwynivere A. Davies, Christopher Strader, Richa Chibbar, Stefania Papatheodorou, Adam A. Dmytriw

**Affiliations:** 1 Department of Oncology, Juravinski Cancer Centre- Hamilton Health Sciences, Hamilton, ON Canada; 2 Department of Surgery, University of Massachusetts Medical Center, Worchester, MA Canada; 3 Digestive Disease Center, Beth Israel Lahey Health, Harvard Medical School, Boston, MA USA; 4 Department of Epidemiology, Harvard T.H. Chan School of Public Health, Boston, MA USA; 5 Department of Medical Imaging, University of Toronto, Toronto, ON Canada

**Keywords:** Lymphoma, Physical activity, Meta-analysis

## Abstract

**Background:**

The literature suggests an increased risk between anthropometrics including higher body mass index and lymphoma incidence; however, the association with physical activity remains unclear. A systematic review/meta-analysis was therefore performed to examine this association with physical activity (total, recreational or occupational).

**Methods:**

PubMed, Web of Science and Embase were reviewed from inception to October 2019 identifying relevant observational studies. Non-Hodgkin lymphoma (NHL) including subtypes diffuse large B cell lymphoma, follicular lymphoma and chronic lymphocytic leukemia/small lymphocytic lymphoma, and Hodgkin lymphoma (HL) were analyzed. Included studies reported activity, lymphoma cases, effect size and variability measures, and were restricted to human subjects of any age. Data was pooled generating summary relative risk (RR) estimates with 95% confidence intervals (CI) using random-effects models with primary outcome of histologically confirmed incident lymphoma.

**Results:**

One thousand four hundred studies were initially identified with 18 studies (nine cohort, nine case-control) included in final analysis. Comparing highest vs. lowest activity categories was protective for all lymphoma (RR 0.89, 95%CI 0.81–0.98). Sensitivity analysis demonstrated effect persistence within case-control studies (RR 0.82, 95% CI 0.71–0.96), but not cohort studies (RR 0.95, 95%CI 0.84–1.07). Borderline protective effect was seen for NHL (RR 0.92, 95%CI 0.84–1.00), but not HL (RR 0.72, 95%CI 0.50–1.04). Analysis by NHL subtype or gender showed no effect. Dose response analysis demonstrated a protective effect (*p* = 0.034) with a 1% risk reduction per 3 MET hours/week (RR 0.99, 95%CI 0.98–1.00).

**Conclusions:**

Physical activity may have a protective effect against lymphoma development; further studies are required to generate recommendations regarding health policy.

**Trial registration:**

This study was registered prospectively at PROSPERO: CRD42020156242.

## Background

Lymphoma is a common malignancy, with non-Hodgkin lymphoma (NHL) incidence estimated at 19.6 per 100,000 people per year and a lifetime risk of 2.2% [1, 2]. By comparison, Hodgkin lymphoma (HL) is much less common with only 8110 new cases and 1000 deaths per year. The incidence for both types has been decreasing over the past decade, with a drop of 0.9 and 1.8% yearly for NHL and HL respectively [3]. While a reassuring trend, up to one-third of patients will still die from NHL within the first 5 years (5-year survival 72%) [4].

In addition to new oncologic therapies, further research is needed to direct disease prevention, specifically modifiable risk factors, such as physical activity. Prior meta-analyses demonstrated a relationship between obesity and lymphoma incidence in a dose-dependent manner (obesity vs. overweight vs. normal weight) [5–7]; however, it remains unclear if there is an association between physical activity and lymphoma independent of body mass index (BMI) [8]. Additionally, BMI does not always reflect obesity or inactivity, therefore an alternate metric was considered. Studies have shown a reduction in the risk of development of colon, breast, and endometrial cancer with physical activity [9], whereas various studies in lung, prostate and ovarian cancer have been inconclusive [10]. Better understanding of the available evidence for lymphoma could inform discussions with patients and better guide health policy recommendations.

Compared to the costs of treatment [11], physical activity is not only cost-effective [12] but is also associated with increased life expectancy and additional health benefits, including reductions in heart disease, hypertension, and diabetes [13]. Unfortunately, adherence to weight loss strategies and caloric restriction is low [13]. Therefore, a demonstrated protective effect against lymphoma and other cancers, independent of weight loss or normal range BMI, could lead to a focus in public health policy on physical activity with downstream effects on overall incidence and survival. This could additionally result in positive health system economic impacts.

This meta-analysis expands on prior work [5, 10, 14] by incorporating two case-control [15, 16] and two cohort studies [17, 18] with 1432 additional lymphoma cases that have since been published, increasing case sample size by approximately 12%. In addition, within previous efforts [10] the outcome key words of “(lymphoma OR Hodgkin)” were likely satisfactory, however the exposure keywords may have missed studies examining specific forms of activity leading to incomplete evaluation which require further examination in subgroup or sensitivity analyses. Lastly, while the effect size in the original meta-analysis suggested a nonsignificant protective effect (pooled odds ratio (OR) = 0.90, 95% confidence interval (CI): 0.79–1.02, *p* = 0.10) [10], the limited number of studies may have precluded definitive assessment because of inadequate power. A latter analyses demonstrated a protective effect [14], although serious methodological concerns exist regarding subjects being included multiple times to account for different forms of activity (occupational and recreational) within the same analysis.

The objective of this meta-analysis is to examine all existing evidence to determine if physical activity leads to a lower risk for developing lymphoma.

## Methods

### Literature search

Cohort and case-control studies that reported the level or amount of physical activity, either recreational, occupational or total, and incidence of lymphoma development were identified using Medline, Embase and Web of Science using the search terms included in Supplementary Table 1 on October 28, 2019. An updated search was completed on August 2, 2020, which is reflected in the included manuscript and figures. Abstracts were screened by two researchers (two groups- G.D. and A.D, C.S and R.C.) working independently to select for full-text reading. In case of disagreement, discussion with all screening researchers ensued until consensus was reached. No restrictions on publication date were applied. Subsequently, two researchers (same groups) independently performed full-text reading of each article for final selection. Additionally, bibliographies of articles selected for full text review were examined for further studies to include.

### Study selection and assessment of quality

Articles were considered eligible if they contained original data on physical activity and risk of lymphoma. Overall or all lymphoma included patients with both NHL and HL; further subgroup analyses was performed as detailed below and according to NHL subtype. Studies were reviewed to ensure no duplication of included patients. If multiple publications utilizing the same population were identified, the most recent and more detailed article was included. The nine-star Newcastle-Ottawa scale (NOS) was used to assess for quality given the observational nature of cohort and case-control studies. G.D. and A.D. independently assessed study quality and group discussion was utilized to resolve disagreements.

### Data extraction

General study characteristics (such as author name, year of publication, number of patients and years of follow up (cohort) or number of cases and controls (case-control)) were collected along with subject characteristics and activity level exposure. Lymphoma was commonly identified through cancer registry linkage, though some studies performed histologic review. As with previous analyses of physical activity and risk for lymphoma, the effect estimate used for pooled analysis compared the highest to lowest or referent physical activity level [10, 14]. In studies presenting multiple models including latency adjustment, only the risk and variability estimate from the maximally adjusted model were included.

### Statistical analyses

Analysis was first conducted including patients from all studies, with risk estimates selected according to the most representative form of physical activity (total, recreational or if neither was available, occupational activity). Relative risk (RR) was chosen as the common measure; hazard ratios (HR) and OR were considered as RR due to the low incidence of lymphoma. Summary estimation of risk was derived by random effects models with associated 95% CI. Estimates were then pooled according to pre-specified subgroups, including: NHL vs. HL, type of physical activity (occupational vs. recreational), gender, and lymphoma subtype including follicular lymphoma (FL), diffuse large B cell lymphoma (DLBCL), and chronic lymphocytic leukemia/small lymphocytic lymphoma (CLL/SLL). Planned subgroup analysis that could not be completed included exposure reporting (self-reported vs. recorded) due to a lack of studies utilizing the latter method, location due to limited studies outside of North America, and age as this was generally included as a covariate. Study heterogeneity was analyzed using the Cochrane’s Q test and the I^2^ statistic utilizing the following cut-offs: < 25% (low heterogeneity), 25–50% (moderate heterogeneity), > 50% (high heterogeneity). Begg’s rank correlation and Egger’s linear regression tests were used to examine for publication bias. Cumulative meta analysis assessed for change in effect size with time, especially as case definitions changed. Meta regression influence analysis was performed to assess for single study effects. Prediction intervals, demonstrating plausible ranges for the effect size in future studies, are included in Forest plots.

Dose response analysis utilized studies that reported metabolic equivalent of task (MET) hours/week of recreational physical activity (seven studies- four cohort, three case control). In studies where men and women were reported only separately, these were included as unique estimates. Midpoints of each range were used as the corresponding “dose” and open ended categories utilized the lower end of the range multiplied by 1.2 as has been previously suggested [19, 20]. Planned analyses were submitted to PROSPERO prior to data extraction. PRISMA Checklist was followed and completed. All statistical analyses were performed using Stata version 15.1 (STATA Corp, College Station, TX, USA). *p* values were two sided, and level of significance was set at < 0.05.

## Results

### Literature search and study characteristics

**Fig. 1:**
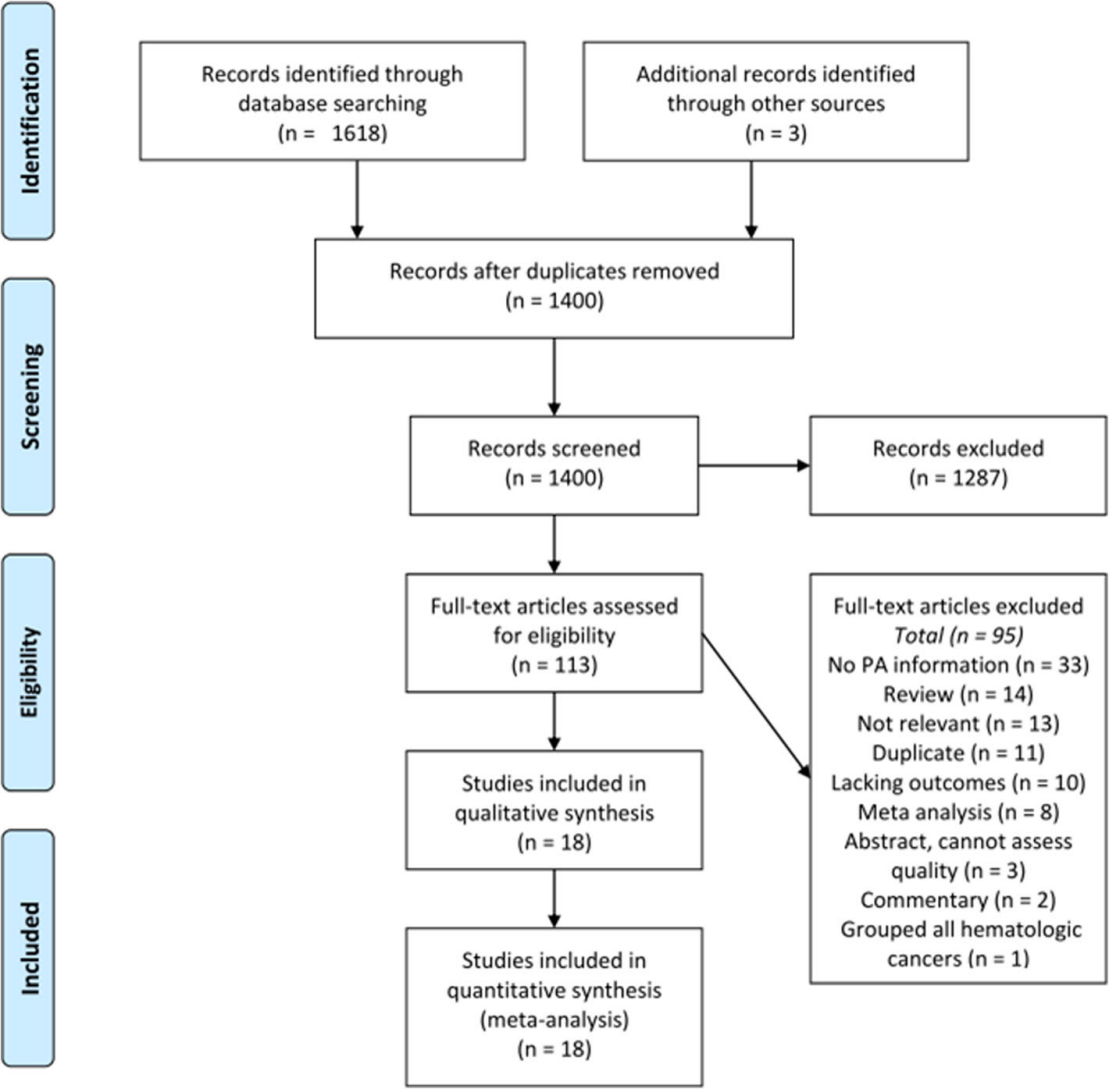
PRISMA flow diagram

Using our search strategy, 1400 potentially relevant studies were identified after removal of duplicates. After title and abstract screening, 113 studies were identified for full text review including those identified on review of prior meta-analyses [10, 14, 21]. Final analysis included 18 studies- 9 cohort [17, 18, 22–28] and 9 case-control [15, 16, 29–35], reporting a total of 12,053 new lymphoma cases. Study characteristics are summarized in Table 1. The PRISMA flowchart detailing study review is presented in Fig. 1; the most common reasons for exclusion were lack of physical activity information (*n* = 33), review or commentary (*n* = 16), not relevant (*n* = 12, ie. physical activity levels after diagnosis of lymphoma), duplicate (*n* = 11), meta-analyses (*n* = 8), etc. Initially, the InterLymph studies were included, comprising a multinational consortium of NHL cases from 19 case-control studies presented as patient level data, however on close review of the datasets there was significant concern for patient overlap so they were excluded [36–39]. Additional review identified the Physical Activity Collaboration of the National Cancer Institute’s Cohort Consortium, which pooled data from 12 prospective cohort studies comprising 1.44 million participants [40]. This evaluated leisure time physical activity and risk for cancer development in cohorts not specifically examining lymphoma development, identifying 6953 incident cases of NHL. However, four of these cohorts have already been included in our initial meta-analysis (accounting for 5643 cases); inclusion of risk estimates from the remaining cohorts (1310 new cases of NHL) did not change our risk estimate (RR 0.90 (0.83–0.98)) and as we were unable to assess quality of individual studies, these numbers/cohorts were not included in our final analysis. The overall pooled HR 0.91 (0.83–1.00) is reassuringly similar to our identified risk estimate.

**Table 1:** Characteristics of included studies according to report type

Authors, year, gender	Study Region	Case number Controls or cohort values	Median age, years (SD)	Lymphoma subtype	Physical activity domain, timing, definition of high vs. low PA	RR/OR/HR (95% CI), high vs. low PA	Adjustment Factors
**Cohort Studies**							
Barberio et al., 2018 [17] Men and women combined	Canada (North America)	91 (NHL) 285,787 person-years follow up	M: 57.8 (8.2) W: 55.9 (9.0) *all cancer cases	NHL	Total, recent (201.2–535.3 vs. 0–113.7 MET hours/wk)	HR 0.73 (0.40–1.36)	Age, sex, ethnicity (white/other), marital status, education level, total household income, geographic area of residence (urban/rural), smoking status, mean alcohol intake (g/d), mean energy intake (kCal/day), self-reported BMI, history of cardiovascular or respiratory condition, family history of cancer (breast or endometrial if female), other PA
					Recreational, recent (35–255.3 vs. 0–7.4 MET hours/wk)	HR 0.86 (0.45–1.65)	
					Occupational, recent (116.8–463.4 vs. 0–34.6 MET hours/wk)	HR 0.68 (0.35–1.31)	
Cerhan et al., 2002 [22] Women	USA (North America)	261 (NHL) 137 (DLBCL) 58 (FL) 37,932 cohort 449,801 person years of follow up	61.7 (no SD reported)	NHL DLBCL FL	Recreational, recent (High vs. low)	NHL: RR 0.84 (0.62–1.15)	Age
						DLBCL: RR 1.0 (0.66–1.52)	
						FL: RR 0.58 (0.29–0.18)	
Kabat et al., 2012 [23] Women	USA (North America)	1123 (NHL) 302 (DLBCL) 214 (FL) 298 (CLL/SLL) 157,852 cohort	Cases: 65.2 (6.9) Non-cases 63.2 (7.2)	NHL DLBCL FL CLL/SLL	Recreational, recent (≥17.5 vs. < 1.6 MET hours/wk)	NHL: HR 1.17 (0.98–1.39)	Age (continuous), alcohol intake, smoking (pack-years), caloric intake, education level, race/ethnicity, BMI, enrollment in OS/treatment arm assignment
						DLBCL: HR 1.03 (0.73–1.45)	
						FL: HR 1.22 (0.83–1.80)	
						CLL/SLL: HR 1.18 (0.84–1.66)	
Lim et al., 2007 [24] Men and women combined	USA (North America)	1381 (NHL) 58 (HL) 472,545 non-cases	60.9–62.5 depending on covariate classification	NHL DLBCL FL CLL/SLL T cell HL	Recreational, recent (NHL ≥5 vs. < 1x/wk.; HL 3- ≥ 5 vs. < 1/wk. activities ≥20 min with increased heart rate and sweating)	NHL: RR 0.97 (0.81–1.16)	Age, race, education, BMI, caloric intake
						DLBCL: RR 0.87 (0.61–1.25)	
						FL: RR 0.96 (0.63–1.46)	
						CLL/SLL: RR 0.80 (0.51–1.25)	
						T-cell: RR 1.25 (0.62–2.49)	
						HL: RR 1.60 (0.72–3.54)	
Lu et al., 2009 [25] Women	USA (North America)	574 (NHL) 155 (DLBCL) 121 (FL) 124 (CLL/SLL) 120,642 cohort	Cohort entry: 52.7 Diagnosis: 69.5	NHL DLBCL FL CLL/SLL	Recreational, lifetime	NHL: RR 1.00 (0.78–1.29)	Height, weight at cohort entry, age at menarche; recent PA also adjusted for long-term strenuous and moderate exercise
						DLBCL: RR 0.97 (0.60–1.55)	
						FL: RR 1.29 (0.69–2.41)	
						CLL/SLL: RR 0.94 (0.55–1.60)	
					Recreational, Recent (≥4 vs. 0–0.5 h/wk./year of strenuous activity)	NHL: RR 1.11 (0.86–1.44)	
						DLBCL: RR 1.00 (0.62–1.62)	
						FL: RR 1.01 (0.57–1.79)	
						CLL/SLL: RR 1.50 (0.86–2.63)	
Pukkala et al., 2000 [26] Men	Finland (Europe)	6 (NHL) 3 (HL) 53,501 person years follow up	Modal age category 45–59 years	NHL HL	Recreational, lifetime (Athletes vs. Finnish population)	NHL: SIR 0.78 (0.29–1.70)	None
						HL: SIR 1.33 (0.27–3.88)	
Teras et al., 2012 [27] Men Women	USA (North America)	2002 (NHL) 435 (DLBCL) 280 (FL) 482 (CLL/SLL) 326 (B-NHL) 110 (T-NHL) 26 (NHL NOS) 184,190 cohort	62.9 (6.4)	NHL DLBCL FL, CLL/SLL B-NHL T-NHL NHL NOS	Recreational, recent (≥17.5 vs. 0 MET hours/wk)	NHL- M: HR 1.02 (0.82–1.26) W: HR 0.69 (0.54–0.89)	Age at baseline, family history of hematopoietic cancer, education, smoking status, alcohol intake, height, BMI, sitting time
						DLBCL- M: HR 1.14 (0.70–1.88) W: HR 0.71 (0.44–1.16)	
						CLL/SLL-M: HR 0.95 (0.62–1.46) W: HR 0.73 (0.44–1.20)	
						FL- M: HR 0.86 (0.49–1.51) W: HR 0.77 (0.41–1.43)	
Van Veldhoven et al., 2011 [28] Men Women Men and women combined	Europe	778 (NHL) 94 (DLBCL) 98 (FL) 142 (B-CLL) 109 (B-NOS) 521,457 cohort	Age at recruitment: 52.9 (9.8) men, 51.0 (10.9) women	NHL DLBCL FL B-CLL B-NOS	Total, recent (Active vs. inactive)	NHL- M: HR 0.83 (0.31–2.24) W: HR 1.50 (0.45–5)	Hypertension, hyperlipidemia, education, diabetes; age and centre stratified *No adjustment for subtypes of NHL
						DLBCL- M: HR 2.03 (0.38–10.79) W: HR 5.03 (1.19–21.33)	
						FL- M: HR 2.82 (0.52–15.23) W: HR 2.59 (0.54–12.38)	
						B-CLL- M: HR 0.76 (0.23–2.53) W: HR 10.95 (1.55–77.48)	
						B-NOS- M: HR 0.16 (0.01–1.95) W: HR 1.60 (0.20–12.52)	
					Occupational, recent (Manual/heavy labor vs. sedentary)	NHL- M: HR 1.05 (0.46–2.41) W: HR 2.06 (0.65–6.51)	
					Recreational, recent (≥45.75 vs. < 14.25 MET hours/wk)	NHL- M: HR 1.11 (0.49–2.52) W: HR 1.25 (0.49–3.16)	
Walter et al., 2013 [18] Men and women combined	USA (North America)	441 (Mature B cell) 104 (CLL) 257 (Other mature B cell) 22 (HL) 65,322 cohort	61.5 (7.4)	Mature B cell neoplasms (including plasma cell disorders) CLL Other mature B cell	Recreational, lifetime (> 4.8 vs. 0.2–1.8 MET hours/wk)	*not reported for mature B cell	Age, sex, race/ethnicity, education, smoking, BMI at baseline, fatigue/lack of energy, self reported health, daily fruit consumption, daily vegetable consumption, history of anemia in year before baseline, family history of leukemia or lymphoma
						CLL: HR 0.52 (0.26–1.03)	
						Other mature B cell: HR 0.80 (0.53–1.22)	
**Case- Control Studies**							
Boyle et al., 2015 [15] Men Women Men and women combined	Canada (North America)	749 (NHL) 202 (DLBCL) 188 (FL) 268 (Other B cell) 70 (T cell) 818 controls	Modal age category 70+ years (cases), 60–69 years (controls)	NHL DLBCL FL Other B cell T cell	Total non-occupational, lifetime (≥85 vs. 0–35.9 MET hours/wk)	NHL- M/W: OR 1.16 (0.87–1.55) M: OR 1.14 (0.78–1.68) W: OR 1.07 (0.68–1.68)	Age group, sex, residential location, ethnicity
						DLBCL: OR 1.22 (0.79–1.88)	
						FL: OR 1.26 (0.80–1.99)	
						Other B cell: OR 1.04 (0.69–1.57)	
						T cell: OR 1.19 (0.61–2.32)	
Brownson et al., 1991 [29] Men	USA (North America)	536 (NHL) 16,611 controls	Not reported	NHL	Occupational, recent (> 80% vs. < 20% active at work)	OR 0.83 (0.56–1.25)	Age, smoking
Cerhan et al., 2005 [30] Men and women combined	USA (North America)	562 (NHL) 32 (DLBCL) 24 (FL) 468 controls* *Population with PA data (Total cases 1321, controls 1057 in study)	Cases: 56.1 years (12.9) Controls: 59.7 years (11.7)	NHL DLBCL FL	Occupational, lifetime (Mostly exercise vs. mostly sit at work)	NHL: OR 0.98 (0.55–1.74)	Age, sex, race and study center, BMI, height
						DLBCL: OR 1.32 (0.63–2.76)	
						FL: OR 1.04 (0.45–2.37)	
					Non-occupational, lifetime (> 1080 vs. 0 MET hours/wk)	NHL: OR 0.83 (0.50–1.38)	
						DLBCL: OR 0.82 (0.41–1.66)	
						FL: OR 0.85 (0.39–1.83)	
Etter et al., 2018 [16] Men Women	USA (North America)	236 (NHL) 87 (HL) Controls 952 (NHL); 348 (HL)	NHL: 56.31 (15.42)	NHL HL	Recreational, lifetime (≥1 vs. < 1 exercise session/wk)	NHL: OR 0.74 (0.55–0.99)	Age, sex, BMI, family history of lymphoma, smoking (pack-years), education level (HL only)
			HL: 32.90 (10.59)			HL: OR 0.53 (0.32–0.87)	
Keegan et al., 2006 [31] Women	USA (North America)	312 (HL) 325 controls	35 (SD not reported)	HL	Recreational, lifetime	19–44 years: OR 0.71 (0.48–1.05)	Age, race/ethnicity, educational level, Jewish upbringing, smoking status 1 year before diagnosis/interview, nursed children, number of miscarriages, having a first or second degree relative hx of lymphoma, Jewish heritage, single room at age 11, living in a single family home at age 8 years, living in a rented family home at age 8 years
						45–79 years: OR 0.35 (0.09–1.36)	
					Recreational, recent (≥2 vs. < 2 strenuous activities/wk. for > 1 month)	19–44 years: OR 0.58 (0.39–0.87)	
						45–79 years: OR 0.45 (0.19–1.06)	
Kelly et al., 2012 [32] Men Women Men and women combined	USA (North America)	950 (NHL) 181 (DLBCL) 242 (FL) 302 (CLL/SLL) 1146 controls	63 (SD not reported)	NHL FL CLL/SLL DLBCL	Recreational, recent (> 45 vs. 1.08 MET hours/wk) *also reported recreational, early adulthood (not listed)	NHL: M/W- OR 1.03 (0.80–1.33) M- OR 1.37 (0.98–1.92) W- OR 0.71 (0.47–1.07)	Age at enrollment, gender and county of residence
						DLBCL: M/W- OR 0.99 (0.62–1.59) M- OR 1.19 (0.64–2.21) W- OR 0.80 (0.38–1.69)	
						FL: M/W- OR 0.98 (0.65–1.48) M- OR 0.96 (0.53–1.72) W- OR 0.90 (0.49–1.67)	
						CLL: M/W- OR 1.09 (0.75–1.58) M- OR 1.71 (1.08–2.70) W- OR 0.53 (0.27–1.06)	
Pan et al., 2005 [33] Men Women Men and women combined	Canada (North America)	1030 (NHL) 419 (DLBCL) 242 (FL) 100 (SLL) 269 (NHL NOS) 3106 controls	Men- Cases: 56.7 (12.5) Controls: 57.9 (14.6)	NHL DLBCL FL SLL NHL Not otherwise specified (NOS)	Recreational, recent (≥34.4 vs. < 6.3 MET hours/wk)	NHL: M- OR 0.79 (0.59–1.05) W- OR 0.59 (0.42–0.81)	Age, province of residence, education, alcohol consumption, pack-years of smoking, total calorie intake, self-reported exposure to some chemicals, ever employment in some occupations, BMI, total calorie intake
			Women- Cases: 57.1 (12.6) 56.2 (12.2)			DLBCL: OR 0.84 (0.62–1.13)	
						FL: OR 0.64 (0.42–0.97)	
						SLL: OR 0.74 (0.41–1.33)	
						NHL NOS: OR 0.64 (0.45–0.92)	
Parent et al., 2011 [34] Men	Canada (North America)	215 (NHL) 54 (HL) 533 controls	Cases: 58.9 (8.01) Controls: 59.6 (7.91)	NHL HL	Occupational, recent (≥75% active vs. ≥75% sedentary at work)	NHL: OR 0.56 (0.26–1.20) HL: OR 0.84 (0.17–4.23)	Age, ethnicity, educational level, socioeconomic status, respondent status, BMI, smoking, other PA
					Recreational, recent (≥1 vs. <1x/wk. sports or activity)	NHL: OR 1.17 (0.81–1.69) HL: OR 0.97 (0.45–2.08)	
					Overall, recent (Intermediate activity at work + recreational PA or high PA at work vs. other)	NHL: OR 0.85 (0.60–1.21) HL: OR 1.20 (0.58–2.47)	
Zahm et al., 1999 [35] Men Women	USA (North America)	1177 (NHL) 3625 control	Median not reported. Modal age category 45–84 years (cases), 75+ years (1162 controls)	NHL	Occupational, lifetime or “usual” occupation (M > 12 vs. < 8; W > 8 vs. < 5 k Joules/min)	M: OR 1.0 (0.7–1.3) W: OR 1.7 (0.2–11.5)	Age, state of residence

**PA Physical activity, *M* Men, *W* Women, *M/W* Men/Women Combined, *NHL* Non-Hodgkin lymphoma, *DLBCL* Diffuse large B cell lymphoma, *FL* Follicular lymphoma, *CLL/SLL* Chronic lymphocytic leukemia/small lymphocytic lymphoma, *NOS* Not otherwise specified, *HL* Hodgkin lymphoma, *BMI* Body mass index

Publication years ranged from 1991 to 2018, and most studies were conducted in North America, with the exception of Pukkala et al. (2000) and van Veldhoven et al. (2011) [26, 28] conducted in Finland and multiple European countries respectively. Four studies reported only effect sizes for women [22, 23, 25, 31], three for men [26, 29, 34], and three studies only presented both genders separately [27, 33, 35]. The remaining studies reported effect estimates for men and women combined +/− separately. Participants ranged from 19 to 84 years of age. Exposure (ie. type, intensity and frequency of activity) was most commonly self- reported by questionnaire, followed by phone and in person interview. Activity period ranged from “current” to lifetime, and four studies reported multiple types of physical activity (total, recreational or occupational) [17, 27, 28, 30]. There was wide variation in physical activity type (total, occupational, recreational) and reporting of activity level (days per week, percentage of time spent on strenuous activity, MET hours per week, etc).

Given the time periods included there were multiple lymphoma classification systems used. For instance, CLL was considered leukemia in the earlier studies, however was later combined with SLL under NHL in a WHO update, thus potentially biasing against an effect (less “lymphoma” cases in the initial studies). Adjustment for confounders varied widely across studies, with most adjusting for age, gender, and several for BMI, other types of activity, or other anthropometric measures. Studies ranged from high risk (three stars) to low risk of bias (eight stars), with the majority of case-control and cohort studies being of moderate (6/9) and low risk (5/9) of bias, respectively (Table 2).

**Table 2:** Quality assessment of included studies

Study	Selection	Comparability	Outcome or Exposure	Total	Risk of Bias
**Cohort Studies**					
Barberio et al., 2018 [17]	***	**	**	7	Low
Cerhan et al., 2002 [22]	***	*	**	6	Moderate
Kabat et al., 2012 [23]	***	**	***	8	Low
Lim et al., 2007 [24]	***	**	***	8	Low
Lu et al., 2009 [25]	**	*	**	5	Moderate
Pukkala et al., 2000 [26]	*		**	3	High
Teras et al., 2012 [27]	***	**	***	8	Low
van Veldhoven et al., 2011 [28]	**	*	*	4	Moderate
Walter et al., 2013 [18]	***	**	***	8	Low
**Case-Control Studies**					
Boyle et al., 2015 [15]	***	**	*	6	Moderate
Brownson et al., 1991 [29]	*	*	*	3	High
Cerhan et al., 2005 [30]	**	**	*	5	Moderate
Etter et al., 2018 [16]	*	**	**	5	Moderate
Keegan et al., 2006 [31]	****	**	*	7	Low
Kelly et al., 2012 [32]		**	**	4	Moderate
Pan et al., 2005 [33]	****	**	**	8	Low
Parent et al., 2011 [34]	**	**	*	5	Moderate
Zahm et al., 1999 [35]	***	*	*	5	Moderate

### New cases of NHL and HL combined (“all lymphoma”)

**Fig. 2:**
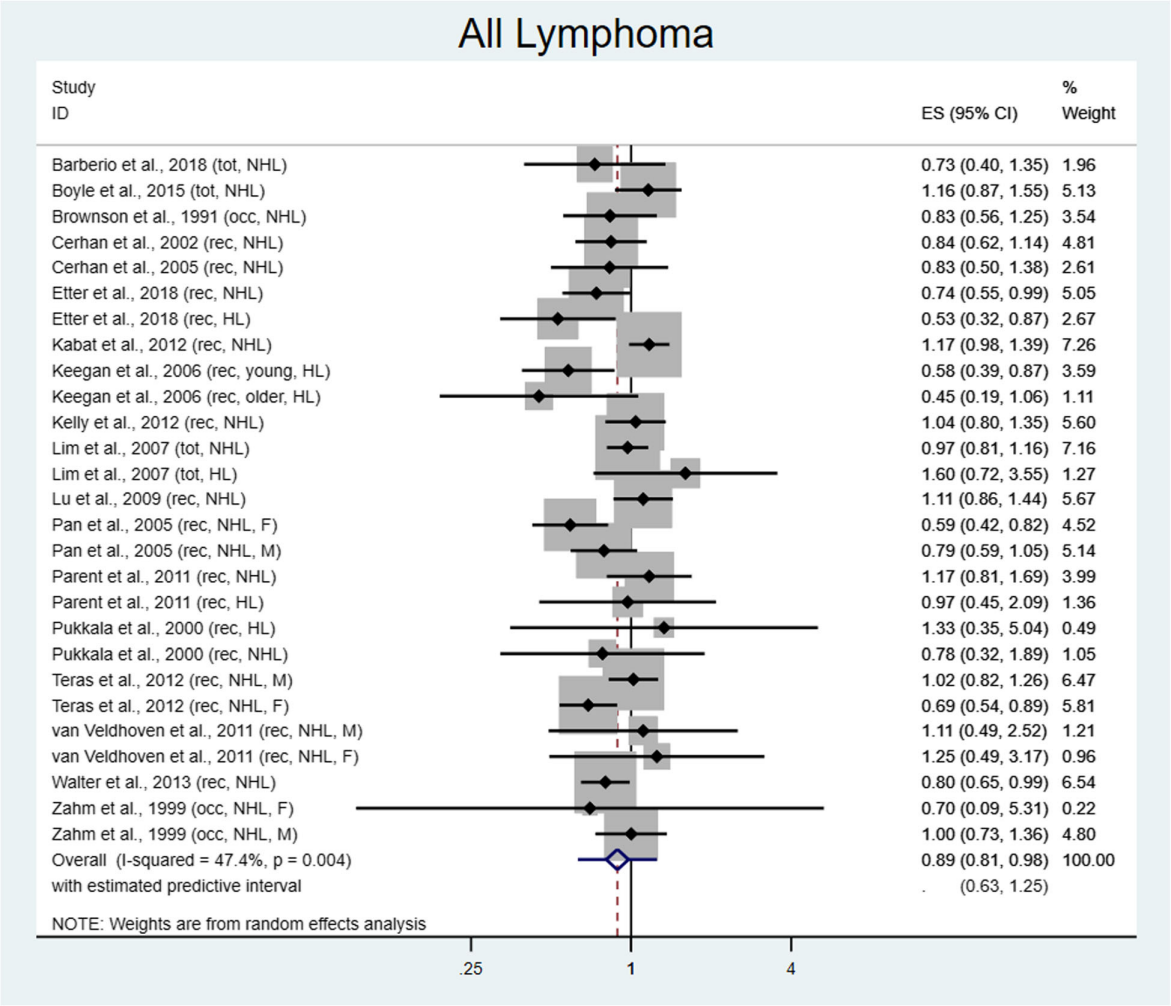
Risk for all lymphoma comparing the highest versus lowest level of physical activity. ES = Effect size

In total, 3009 new cases of lymphoma were diagnosed within the highest physical activity level. This yielded 27 estimates comparing the highest to lowest level of physical activity, yielding a protective summary RR of 0.89 (95% CI 0.81–0.98) (Fig. 2). The Q statistic indicates moderate heterogeneity of study results (chi2 = 49.39; I^2^ = 47.4%, *p* = 0.004). Use of Egger’s test demonstrates no small study effects (*p* = 0.29) and influence analysis excluding one study at a time shows slight variation in effect size, but this does not change by more than 2%. The confidence intervals do not include 1 with exclusion of any studies. Sensitivity analysis utilizing cumulative meta-analysis demonstrates stabilization of effect size around 2009.

### Sensitivity analysis by study type

Sensitivity analysis was performed by analyzing cohort and case-control studies separately assessing “all lymphoma”. Among the nine cohort studies, 13 effect estimates were included as two studies described effect estimates separately by gender only and one study included both NHL and HL separately. The summary RR was not significant at 0.95 (95% CI 0.84–1.07; I^2^ = 39.9%, *p* = 0.07). By comparison, among the nine case-control studies, there were 14 effect estimates (both NHL and HL estimates from two studies, two studies with genders reported separately and one study with two individual age groups), and demonstrated significant protection with higher levels of exercise at RR 0.82 (95% CI 0.71–0.96; I^2^ = 49.5%, *p* = 0.02).

### Subgroup analysis

**Fig. 3:**
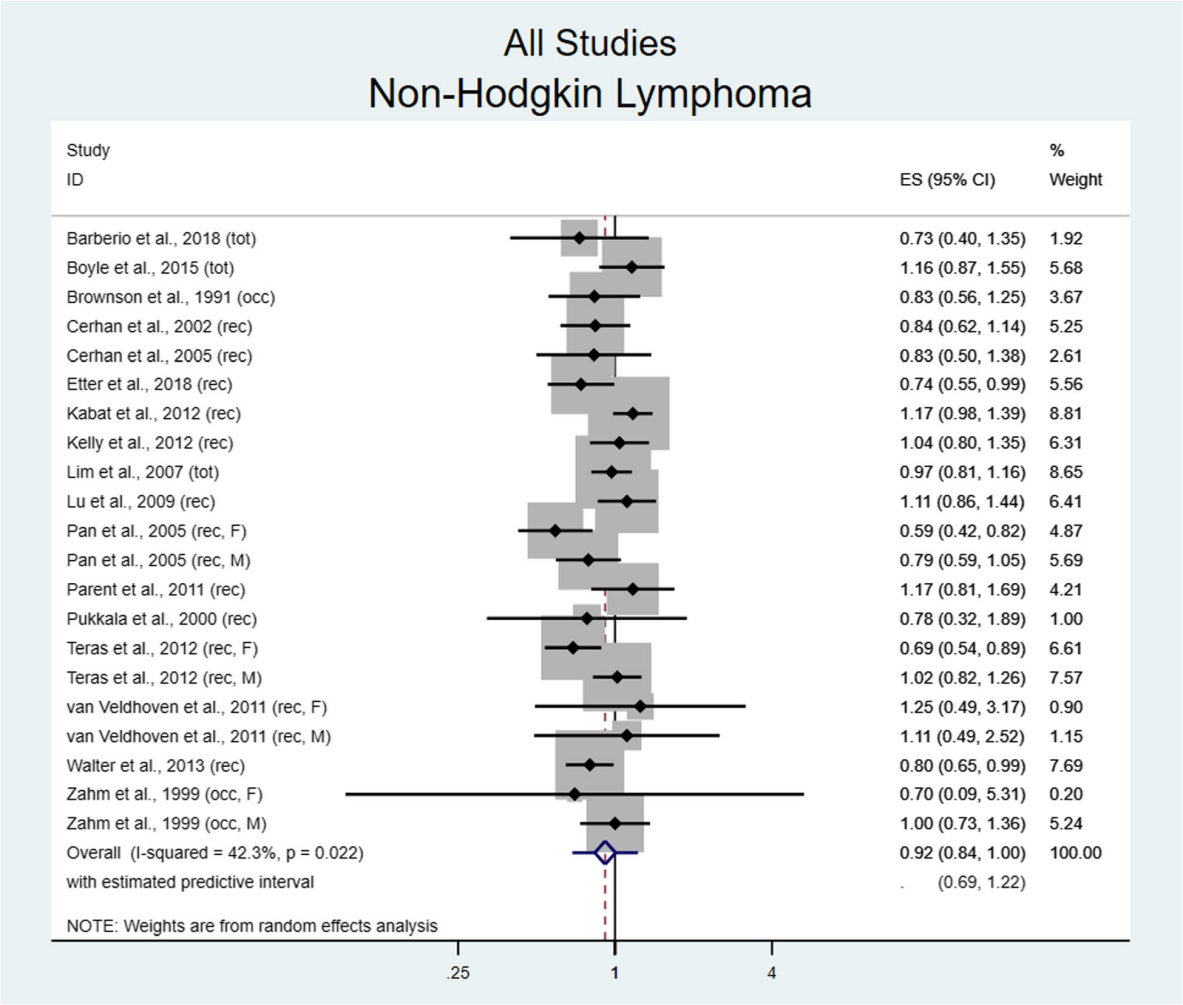
Risk for non-Hodgkin lymphoma comparing the highest versus lowest level of physical activity. ES = Effect size

Seventeen of 18 studies examined the risk for NHL development in relation to physical activity. Pooling of effect size showed a marginally significant protective effect between the highest and lowest levels of physical activity (RR 0.92, 95% CI 0.84–1.00, I^2^ = 42.3%, p = 0.02) seen in Fig. 3. No benefit was found in males with NHL (RR 1.02, 95% CI 0.90–1.15; I^2^ = 5.2%, *p* = 0.39), while in females there was a non-significant trend towards benefit (RR 0.88, 95% CI 0.72–1.08; I^2^ = 66.2%, *p* = 0.003). Interestingly, utilizing sex specific risk estimates significantly reduced between study heterogeneity as demonstrated by Cochrane’s Q test (chi2 8.44) and I^2^ for men (see previous), but did not reduce heterogeneity for women, potentially suggesting differences in population selection.

Subgroup analysis by NHL subtypes of DLBCL, FL and CLL/SLL was performed to assess for biological heterogeneity in response to physical activity. In studies reporting DLBCL incidence, no difference in risk was found (RR 0.95, 95% CI 0.83–1.09; I^2^ = 0%, *p* = 0.44). This was consistent when restricting to estimates in men (RR 1.27, 95% CI 0.71–2.27; I^2^ = 0%, *p* = 0.56) or women (RR 0.97, 95% CI 0.76–1.25; I^2^ = 28.6%, *p* = 0.22).

Among those studies examining FL, no protective effect was found with physical activity (RR 0.95, 95% CI 0.80–1.12; I^2^ = 10.4%, *p* = 0.34) in both genders, or when limited to men (RR 0.96, 95% CI 0.65–1.43; I^2^ = 0%, *p* = 0.42) or women (RR 0.97, 95% CI 0.75–1.26; I^2^ = 9.3%, *p* = 0.36) though sex stratified estimates were limited to only three and five studies, respectively.

Lastly, in studies assessing CLL/SLL incidence, there was no benefit between the highest and lowest levels of physical activity (RR 0.95, 95% CI 0.76–1.20; I^2^ = 43.6%, *p* = 0.07), or according to sex specific estimates for men (RR 1.18, 95% CI 0.73–1.91; I^2^ = 49.9%, *p* = 0.14) or women (RR 1.07, 95% CI 0.65–1.74; I^2^ = 70.1%, *p* = 0.010, suggesting high heterogeneity).

**Fig. 4:**
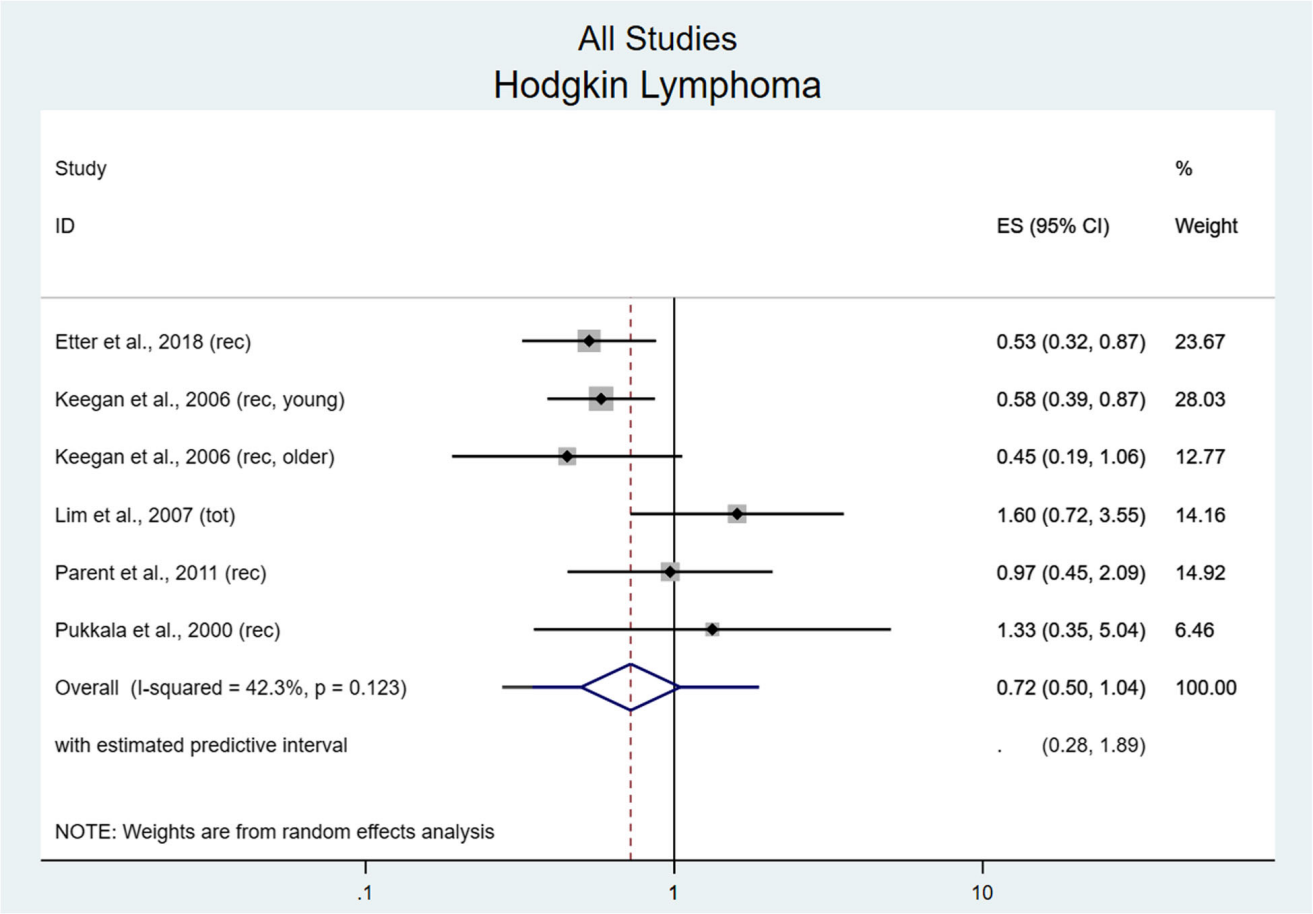
Risk for Hodgkin lymphoma comparing the highest versus lowest level of physical activity. ES = Effect size

In total, five studies reported HL incidence yielding six effect estimates (one study reported estimates for two separate age groups). There was a trend towards protective benefit comparing highest and lowest levels of physical activity (RR 0.72, 95% CI 0.50–1.04; I^2^ = 42.3%, *p* = 0.12) that was not significant (Fig. 4). A significant benefit was found in women (RR 0.55, 95% CI 0.39–0.80; I^2^ = 0%, *p* = 0.60), but not men (RR 1.05, 95% CI 0.54–2.04; I^2^ = 0%, *p* = 0.69).

**Fig. 5:**
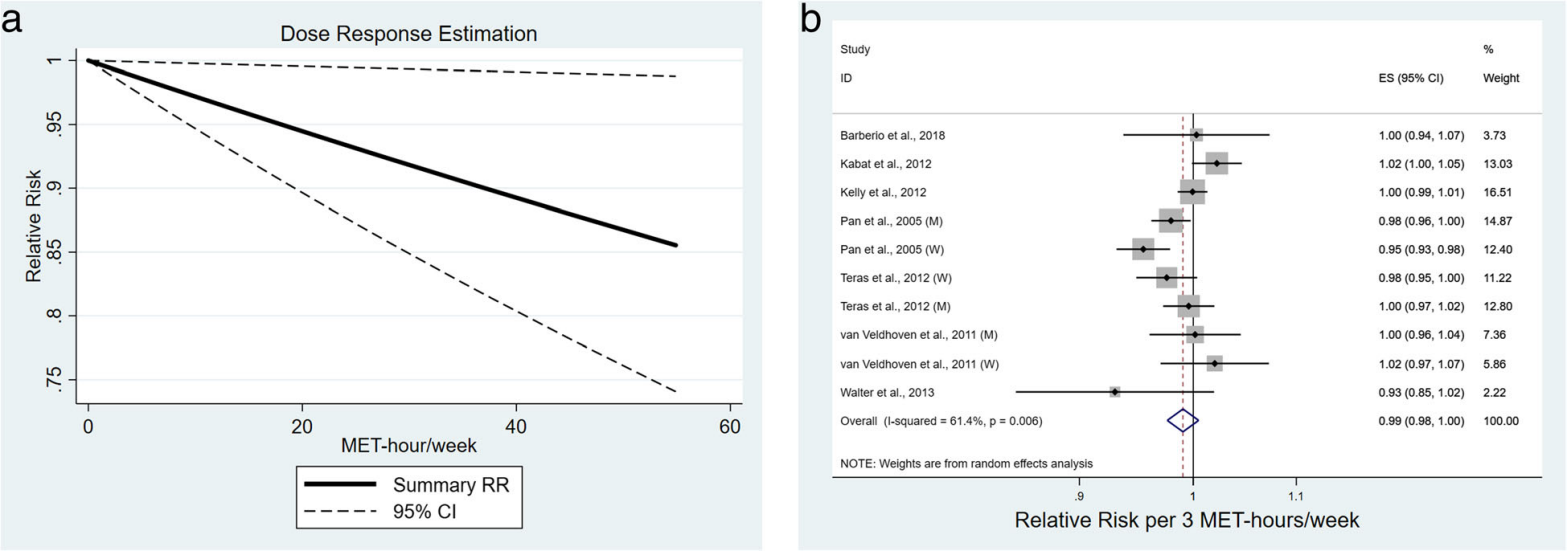
Exploratory dose response analysis for studies reporting recreational physical activity in metabolic equivalent of task (MET) hours per week (**a**), and Forest plot demonstrating summary reduced relative risk per 3 MET hours per week (**b**)

Subgroup analysis of recreational versus occupational activity demonstrated a protective summary value (RR 0.85, 95% CI 0.76–0.96; I^2^ = 53.3%, *p* = 0.002, high heterogeneity) for recreational PA; however, this was not seen for occupational activity (RR 0.93, 95% CI 0.76–1.14, I^2^ = 0%, *p* = 0.75). Total activity level was not analyzed separately as this is generally a composite of the prior two measures.

### Dose response analysis

Exploratory dose response analysis was performed utilizing the seven studies which reported MET hours/week of recreational physical activity and effect estimates. Most studies reported tertiles or quartiles of physical activity, and upper levels ranged from > 4.8 MET hours/week to ≥45.75 MET hours/week, highlighting the differences in activity capture and populations. Dose response comprised 6019 newly diagnosed lymphoma cases and demonstrated a significant protective effect (*p* = 0.03, Fig. 5), estimated as a 1% reduction in lymphoma incidence per 3 MET hours/week of activity (RR 0.99, 95% CI 0.98–1.00; I^2^ = 61.4%, *p* = 0.0003).

## Discussion

The aim of the current meta-analysis was to present an updated review of the literature on the association between physical activity and risk of lymphoma, and to address limitations of previous meta-analyses.

We demonstrate that high physical activity levels have a significant protective effect for the overall risk of lymphoma development, borderline protective effect for NHL, and no effect on HL incidence in both genders combined, though women alone had a reduced risk of lymphoma within the highest level of physical activity. This effect estimate is consistent with previous studies by Vermaete et al. (2013) demonstrating a pooled RR of 0.90 (95% CI 0.79–1.02) and Jochem et al. (2014) with a significant pooled RR of 0.90 (95% CI 0.81–0.99) for lymphoma development [10, 14]. This suggests stabilization of effect with time as multiple newer studies were included in this updated analysis. Physical activity is postulated to reduce risk of other malignancies [41–44] through a variety of mechanisms that may be relevant to clonal lymphocyte dysregulation. These can include: epigenetic modification, such as via restoration of normal methylation status or upregulation of p53 proteins [45]; improved insulin sensitivity with resistance identified as a mediator between increased height or weight and lymphoma risk [21]; reduction of inflammatory cytokines via obesity reduction and lower levels of the ASC protein [45]; and various other biologic etiologies.

Sensitivity analysis demonstrated a non-significant effect in cohort studies, which would have a lower risk of bias due to their prospective design. This is relevant for physical activity, as there is potential for recall bias in self-reported recreational exercise, especially when considering lifetime values. However, this may be less relevant for occupational activity which was usually expertly coded according to an occupation algorithm. Another potential source of bias is reverse causation whereby those reporting lower baseline activity may paradoxically have an undiagnosed cancer. Multiple cohorts addressed this by performing “latency adjusted” analysis, in which cases diagnosed within the first 2 years of study were excluded without significant change in their estimates [17, 23, 25].

No noticeable difference in effect size was seen by NHL subtype, and the small number of further gender stratified studies limits specific conclusions. Several studies reported effect estimates for other NHL subtypes, such as mycosis fungoides/Sezary syndrome, marginal zone lymphoma, peripheral T cell lymphoma, and lymphoplasmacytic lymphoma/Waldenstrom macroglobulinemia, but were too infrequent to synthesize. The latency of lymphoma may be important to consider when deciding on relevant time period of activity. For example, recent BMI has significantly more impact on DLBCL than FL or CLL/SLL [21], which may relate to the longer period of development for indolent lymphomas. Analogously, lifelong activity may be more relevant to these lymphomas, whereas recent activity may have a stronger impact on aggressive lymphoma subtypes. This could be explored in future studies comparing lifelong and recent activity to NHL subtype incidence.

In examining gender modification, we included only studies that reported effect estimates for men or women alone. No protective effect of physical activity was seen for men, however in women there was reduced risk for HL and a protective trend for NHL. The biologic mechanisms for this effect are not fully understood, however most lymphomas are more common in males, thus development may be more heavily influenced by other biologic factors within this gender [37]. Previous authors have suggested this may also be due to estrogen or reproductive effects [14]- potentially by reducing body fat and thus the location of conversion from androstenedione to estrogen or by increasing levels of sex hormone binding globulin [6], which may affect inflammatory cytokine production. These results should be interpreted with caution, given the limited number of studies with sex stratification, although this was frequently controlled for in multivariable risk analysis.

Biologic plausibility is supported by the findings of reduced risk for lymphoma development by increasing MET hours/week of recreational physical activity. This occurred linearly with a reduction of 1% per 3 MET hours/week, equivalent to 1 hour of walking at average pace. Dose response has been demonstrated in other malignancies such as breast cancer [46] and represents an important step towards developing guidelines for activity. The World Health Organization recommends 150 min of moderate (3–6 MET/hour) level physical activity per week for adults, which translates to an average of 11.25 additional MET hours in addition to daily activities [47]. Using our estimated reduction of 1% per 3 MET hours/week, this could result in a relative 3.75% fewer cases of lymphoma with guideline adherence.

Several limitations deserve discussion, primarily the heterogeneity in physical activity reporting. Some studies consider total activity, combining occupational, recreational and occasionally household activities, while others report only occupational or recreational activity, with widely discrepant referent and “highest level” of physical activity. As described previously, the upper quartile of MET hours/week of physical activity could vary by up to 10 between studies, ranging from > 4.8 [18] to ≥45 [32]. Ideally, patient level data would be accessed such that risk could be considered continuously rather than by quartile, however this is impractical given how initial data were collected (written questionnaire, etc) especially given variation in activity “units”. Considerations for future analysis include collapsing all levels of physical activity for comparison to basal inactivity, similar to a meta-analysis examining cancer risk related to pickled vegetable consumption [48].

Additionally, the timing of activity varied greatly with studies reporting “baseline” activity at enrollment, personal quantification of lifetime activity, specific age activity, and so on. Given the overall incidence of lymphoma and non-compliance inherent to activity interventions [13], a randomized study examining this association is impractical; thus, health care policy recommendations will undoubtedly rely on observational studies. Standardization of timing, type and intensity of physical activity within future cohort studies is therefore imperative.

A further weakness includes the type and number of confounders adjusted for. While most studies adjusted for age, the number of included covariates ranged from zero to 12, demonstrating the wide variance in suspected etiologic triggers which was corroborated by the InterLymph consortium [37]. A recent meta-analysis demonstrated a positive association of early adult BMI and weight with NHL risk [21], and the complex interplay between BMI and physical activity was not consistently captured. Control selection in case-control studies is of particular importance, and one study utilized male patients with other cancers which could bias towards the null [29]. Gender stratified analyses were limited, especially for NHL subtypes and preclude definitive conclusions. Additionally, definitions of lymphoma have significantly changed in the past two decades with introduction of the WHO classification in the early 2000s, and therefore there is likely misclassification in older studies.

## Conclusions

Overall, the highest level of physical activity appears to be protective against lymphoma development, compared to the lowest level. While this association is most apparent for NHL, a paucity of studies have assessed HL outcomes likely due to the lower incidence of this malignancy, and thus conclusions are limited. Female subjects appear to benefit from higher levels of activity however the limited number of gender stratified results preclude sex specific conclusions. Dose response analysis supports these conclusions, with a linear decrease in incidence seen with increasing recreational physical activity. This is especially relevant as recreational activity is more modifiable than occupation associated activity. Our study represents a comprehensive review of current literature without significant methodological concerns seen in previous analyses examining this relationship. Further higher quality cohorts, controlling for both anthropometric and cancer risk factors and with standardized activity reporting are needed to reach definitive conclusions and develop policy recommendations.

## Supplementary information

**Additional file 1.**

## Data Availability

All data generated or analysed during the study are included in this published article [and its supplementary information files].

## Abbreviations

NHL: Non-Hodgkin lymphoma
HL: Hodgkin lymphoma
RR: Relative risk
CI: Confidence intervals
BMI: Body mass index
OR: Odds ratio
NOS: Newcastle-Ottawa scale
HR: Hazard ratio
DLBCL: Diffuse large B cell lymphoma
FL: Follicular lymphoma
CLL/SLL: Chronic lymphocytic leukemia/small lymphocytic lymphoma
MET: Metabolic equivalent of task
