# Supplementary material for: The relationship between physical activity and lymphoma: a systematic review and meta analysis
Source: BMC Cancer. 2020 Oct 6;20:962. doi: 10.1186/s12885-020-07431-x (PMC7539461; doi:10.1186/s12885-020-07431-x)
Supplement: Supplementary file 1 — Additional file 1. [file 12885_2020_7431_MOESM1_ESM.docx]

**Supplementary Material for:**

**The Relationship between Physical Activity and Lymphoma: a Systematic Review and Meta Analysis**

**Supplementary Table 1: Search Terms by Database**

| 1. **PubMed** |
| --- |
| (“Exercise”[Mesh] OR “Sports”[mesh] OR exercise[tiab] OR exercising[tiab] OR physical activit*[tiab] OR physical inactivit*[tiab] OR physical fitness[tiab] OR sedentary[tiab] OR "physical exertion"[Mesh]) AND (“Lymphoma”[MESH] OR lymphoma*[tiab] OR Hodgkin*[tiab])  459 results  To enrich for studies that measure risk:  (“Exercise”[Mesh] OR “Sports”[mesh] OR exercise[tiab] OR exercising[tiab] OR physical activit*[tiab] OR physical inactivit*[tiab] OR physical fitness[tiab] OR sedentary[tiab] OR "physical exertion"[Mesh]) AND (“Lymphoma”[MESH] OR lymphoma*[tiab] OR Hodgkin*[tiab]) AND (“Risk Factors”[mesh] OR “Incidence”[mesh] OR risk*[tiab] OR incidence[tiab])  159 results |
| 1. **Embase Session Results** |
| *many terms fall under ‘physical activity, capacity and performance’ including exercise, exercise capacity, physical activity, physical inactivity, training, etc.  *lymphoma includes Hodgkin disease  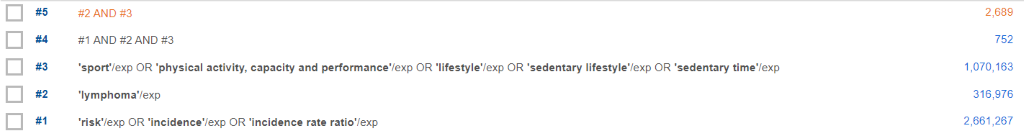  #5: #2 AND #3: 2,689  #4: #1 AND #2 AND #3: 752  #3: 'sport'/exp OR 'physical activity, capacity and performance'/exp OR 'lifestyle'/exp OR 'sedentary lifestyle'/exp OR 'sedentary time'/exp: 1,070,163  #2: 'lymphoma'/exp: 316,976  #1: 'risk'/exp OR 'incidence'/exp OR 'incidence rate ratio'/exp: 2,661,267 |
| 1. **Web of Science** |
| **TOPIC:** (lymphoma OR Hodgkin) *AND* **TOPIC:** ("physical activit*" OR sport* OR sedentary OR exercis* OR "physical fitness")  **Timespan:** All years. **Databases:**  WOS, BCI, BIOSIS, CABI, CCC, DRCI, DIIDW, KJD, MEDLINE, RSCI, SCIELO, ZOOREC.  1,648 results  **TOPIC:**(lymphoma OR Hodgkin) *AND***TOPIC:**("physical activit*" OR sport* OR sedentary OR exercis* OR "physical fitness") *AND***TOPIC:**(risk OR incidence)  **Timespan:**All years.**Databases:**WOS, BCI, BIOSIS, CABI, CCC, DRCI, DIIDW, KJD, MEDLINE, RSCI, SCIELO, ZOOREC. |

**Supplemental Figures.**

**All Lymphoma- Assessment for Heterogeneity, Small Study Effect.**

**Chronological Assessment, Funnel Plot, Egger’s Test.**

**All Lymphoma- Cohort Studies Estimates only**

**All Lymphoma- Cohort- Assessment for Heterogeneity, small study effect.**

**Chronological Assessment, Funnel Plot.**

**All Lymphoma- Case-Control Study estimates only**

**All Lymphoma- Case-Control- Assessment for Heterogeneity, small study effect.**

**Chronological Assessment, Funnel Plot.**

**NHL- All Patient Estimates**

**NHL-Men Alone**

**NHL- Women Alone**

**DLBCL- All Patients**

**DLBCL- Men Alone**

**DLBCL- Women Alone**

**FL- All Patients**

**FL- Men Alone**

**FL- Women Alone**

**CLL/SLL- All Patients**

**CLL/SLL- Men Alone**

**CLL/SLL- Women Alone**

**HL- All Patients**

**HL- Men Alone**

**HL- Women Alone**

**All Lymphoma- Recreational PA**

**All Lymphoma- Occupational RA**
